# Understanding the Role of Dicer in Astrocyte Development

**DOI:** 10.1371/journal.pone.0126667

**Published:** 2015-05-11

**Authors:** Shen-Yi Bruce Howng, Yong Huang, Louis Ptáček, Ying-Hui Fu

**Affiliations:** 1 Department of Neurology, University of California San Francisco, San Francisco, California, United States of America; 2 Howard Hughes Medical Institute, School of Medicine, San Francisco, California, United States of America; Rutgers University, UNITED STATES

## Abstract

The *Dicer1* allele is used to show that microRNAs (miRNAs) play important roles in astrocyte development and functions. While it is known that astrocytes that lack miRNAs are dysregulated, the *in vivo* phenotypes of these astrocytes are not well understood. In this study, we use Aldh1l1-EGFP transgene, a marker of astrocytes, to characterize mouse models with conditional *Dicer1* ablation (via either human or mouse GFAP-Cre). This transgene revealed novel features of the defective astrocytes from the absence of miRNA. Although astrocyte miRNAs were depleted in both lines, we found histological and molecular differences in the Aldh1l1-EGFP cells between the two Cre lines. Aldh1l1-EGFP cells from hGFAP-Cre mutant lines displayed up-regulation of Aldh1l1-EGFP with increased proliferation and a genomic profile that acquired many features of wildtype primary astrocyte cultures. In the young mGFAP-Cre mutant lines we found that Aldh1l1-EGFP cells were disorganized and hyperproliferative in the developing cerebellum. Using the Aldh1l1-EGFP transgene, our work provides new insights into the roles of miRNAs in astrocyte development and the features of astrocytes in these two mouse models.

## Introduction

Conditional *Dicer1* alleles allow researchers to show the importance of miRNAs in developmental processes, including astrocyte development and function [1–4]. While studies have shown that astrocytes lacking miRNAs are dysregulated, the molecular changes that occur to these astrocytes are unclear. In this study, we use the Aldh1l1-EGFP transgene, a recently characterized marker for astrocytes, to characterize the changes to astrocytes in two different mouse models where mature miRNAs are ablated in astrocytes, via hGFAP-Cre or mGFAP-Cre.

MiRNAs are endogenous short hairpin non-coding RNAs that regulate the function and development of cellular processes by inhibiting the synthesis of gene products [5, 6]. *Dicer1* encodes a ribonuclease that cleaves miRNAs into their mature functioning form. Studies have used a conditional *Dicer1* allele to show that the loss of miRNAs in neural precursor cells result in dysregulated brain development and functions [3, 5, 7–10]. Although *Dicer1* is also absent in astrocytes in these models, these studies focused on the effects of losing miRNAs on neuronal differentiation and survival and did not characterize the impact of miRNA depletion on astrocytes [3, 7, 10–12]. When *Dicer1* is ablated in astrocyte precursor cells, some studies have shown that staining of GFAP is altered [3, 4, 9]. The roles of miRNAs in astrocyte functions were further examined in another study using Cre transgenes that were expressed more specifically in astrocytes. In that study, the ablation of *Dicer1* in astrocytes resulted in non-cell autonomous neurodegeneration in the cerebellum [1]. While that study indicated that astrocytes appeared immature at postnatal day 30 (P30), earlier developmental defects of the astrocytes were not assessed. Additionally, in both mouse models, many features of the astrocytes lacking mature miRNAs remain unknown.

Here, we utilized the Aldh1l1-EGFP transgene, a pan-astrocyte marker, to characterize the morphological and molecular phenotypes of astrocytes in the absence of *Dicer1* [13, 14]. We assessed Aldh1l1-EGFP cells in two different mouse models where *Dicer1* was ablated by astrocyte Cre lines. One Cre line expressed before and the other line expressed after astrogliogenesis. We found that Aldh1l1-EGFP cells exhibited distinct dysregulated features. Forebrain Aldh1l1-EGFP cells in the mouse model where *Dicer1* was ablated early (hGFAP-Cre) had features of immature astrocytes and primary astrocytes, whereas forebrain Aldh1l1-EGFP cells in the mouse model where *Dicer1* was ablated later (mGFAP-Cre) did not have obvious defects during development. As previously reported, astrocytes had dysregulation in the developing cerebellum in the mice generated from mGFAP-Cre. In using the Aldh1l1-EGFP transgene, we found additional defects of the astrocytes in the mGFAP-Cre model at an earlier time frame than previously described [1]. The use of Aldh1l1-EGFP transgene allowed us to identify several novel features of astrocytes in mouse models where miRNAs are ablated from astrocytes.

## Materials and Methods

### Mice

BAC Aldh1l1-EGFP transgene were generated by GENSAT. hGFAP-Cre and mGFAP-Cre (line 77.6) lines were obtained from the Jackson laboratory. Mice with conditional *Dicer1* allele were obtained from the McManus lab at UCSF [15]. These conditional *Dicer1* alleles contained lox sites flanking exon 23 which is excised in the presence of Cre. This exon encodes most of the second RNaseIII domain, necessary to convert precursor miRNAs into mature forms when inactivated [15]. mGFAP-Cre *Dicer1* and hGFAP-Cre *Dicer1* experiments were conducted in C57/B6 background and mixed FVB and C57/B6 backgrounds, respectively. Animals of both sexes were used. The care and treatment of the animals were conducted under the strict guidelines of the Guide for the Care and Use of Laboratory Animals of the National Institutes of Health. Animals were anesthetized with 2.5% avertin solution according to guidelines. During euthanasia, mice were first exposed to carbon dioxide followed by cervical dislocation. The protocol was approved by the Institutional Animal Care and Use Committee of the University of California San Francisco (Approval Number: AN089663-01E). Primers for animal genotypes include the following:

hGFAP-CreF-ACTCCTTCATAAAGCCCT

hGFAP-CreR-ATCACTCGTTGCATCGACCG

EGFPForward- CCTACGGCGTGCAGTGCTTCAGC

EGFPReverse- CGGCGAGCTGCACGCTGCCGTCCTC

Dicer1F-CCTGACAGTGACGGTCCAAAG

Dicer1R-CATGACTCTTCAACTCAAACT

wt: 351 bp; floxed allele: 420 bp

mGFAP-CreF-CCGGGCTGCCACGACCAA

mGFAP-CreR-GGCGCGGCAACACCATTTTT

### Immunohistochemistry

To obtain tissues for immunohistochemistry, animals were deeply anesthetized and perfused with 4% PFA in PBS. After dissection, tissues were incubated in fixative for two days and then transferred to 30% sucrose (PBS) solution for another two days. After two days of incubation in 30% sucrose incubation, fixed tissues were frozen in OCT media and sliced to 20 μm sections. For staining, samples were permeabilized in ice cold acetone for 10 minutes. The samples were then washed with PBS before being blocked for 1 hour in room temperature with blocking buffer (5% normal goat serum, NGS, and 0.01% triton-X in PBS). Samples were stained with primary antibodies overnight at 4°C and stained with secondary antibodies for two hours at room temperature the following day. Primary antibodies used were GFAP (Dako, 1/500) and ki67 (Thermo, 1/400). Anti-rabbit secondary fluorochromes conjugated with Cy3 (Jackson ImmunoResearch) were used as secondaries. Slides were mounted with Vectashield (Vector Laboratories). Visualization of the samples was performed with either Leica DMI 6000B or Zeiss Axio imager M1 microscopes. Images were processed with ImageJ (NIH) and Zen (Zeiss) softwares.

### Primary astrocyte cultures

Primary astrocytes were obtained from P2 forebrain. Preparation was performed as described in Cahoy et al. 2008 [16]. Primary cells were used after 2 weeks *in vitro* with a passage at 1 week. For FACS isolation, cells were detached using. 25% Trypsin-EDTA (Life Technologies), then resuspended in Leibovitz L-15 media (Life Technologies) before FACS. After sorting, Trizol (Life Technologies) was added to the cells for subsequent RNA isolation.

### Counting of cells in micrograph images

Counting of cells was performed using ImageJ (NIH). For the brains, 20 μm sections were used and images of cerebral cortex were taken with Leica DMI 6000B. Using ImageJ, areas of 500 μm^2^ unit was marked and numbers of positive cells were counted. The significance levels were calculated using Student’s *t* test, unpaired. Graphs were made using Prism software (GraphPad Software). For mGFAP:CKO cerebellum, ki67 positive cells were counted if they localized between Bergmann glia and pial surface of the cerebellum.

### Cellular preparation of *in vivo* Aldh1l1-EGFP positive cells for FACS

Isolation procedure of the EGFP cells was adapted from Cahoy et al. While we followed the published protocol, we modified the incubation time depending on the ages of the forebrains. P7 forebrains were digested for 25 minutes while P16 and older samples were digested for 80 minutes in papain based digestion media.

### Flow cytometry

EGFP positive cells were sorted as described in Molofsky et al [17]. Arias III (BD Bioscience) machine was used to perform the sorting. For each experiment, samples lacking Aldhl1-EGFP were used as negative control. EGFP positive cells were gated on forward/side scatter, DAPI (Life Technologies) exclusion for live cells, and EGFP signals. Samples were sorted into PBS containing 3% FBS. All samples were re-sorted. After collection of data, samples were analyzed using FlowJo (Tree Star). Once the samples were isolated, the cells were centrifuged for 5 min at 1000 g. The cell pellets were then resuspended in Trizol (Life Technologies) for RNA isolation.

To compare the EGFP intensity of the Aldh1l1-EGFP cells, comparison of the wildtype and mutant cells were always performed on the same day. To determine the differences in number of Aldh1l1-EGFP positive cells, the percentage of EGFP positive cells were determined only from live cell population that were gated for forward/side scatter and excluded DAPI. Representative examples of gating strategies are in S2 Fig, S7 Fig, S9 Fig, S10 Fig, and S11 Fig. We used similar gating strategies for animals of the same age in both hGFAP:CKO and mGFAP:CKO models.

### RNA isolation

Total RNA was isolated using Trizol (Life Technologies) following manufacturer’s protocol. For FACS samples, roughly 10,000 cells were used to isolate total RNA. In the small quantity samples, 250 μg/ml of glycogen was added as a carrier. Samples were precipitated in isopropanol at -20°C for at least two hours. RNA was reconstituted in 50 μl of RNase/DNase free water. Subsequently, the RNA was cleaned with RNA Clean and Concentrator—5 columns (Zymo) and treated with DNase (Life Technologies) in columns following Zymo protocol. 11 μl of RNase/DNase free water was used to elute the RNA. Total RNA was quantified using Agilent Pico Chip (Agilent).

### Microarray

Microarray experiments, which used 2 ng of total RNA, were performed according to manufacturer’s protocol. Samples were amplified using NuGEN Pico V2 (NuGEN) kit and hybridized onto Affymetrix Mouse Gene 1ST chip.

For the generation of the fold change, normalization of the microarray data, gene ontology list, and statistical significance of fold changes, we used the ArrayStar (DNASTAR) analysis program. Normalization was performed using Robust Multi-array Average (RMA) algorithms. Data from 3 samples each of wildtype P7 Aldh1l1-EGFP cells, hGFAP:CKO P7 Aldh1l1-EGFP cells and wildtype primary Aldh1l1-EGFP cells were normalized together. Significance levels were determined by moderated t- test [18]. Given the small sample size that was used, the moderated t-test is better suited at reducing the background noise of the data [18]. False-discovery-rates (FDR) was determined using Benjamini-Hochberg test [19]. Microarray data are available in the ArrayExpress database (www.ebi.ac.uk/arrayexpress) under accession number E-MTAB-2995.

To identify upstream miRNA regulators, we used Ingenuity Pathway Analysis (IPA) to analyze all genes that were differentially up-regulated in mutant samples. As a negative control in the analysis, we used differentially down-regulated genes in the mutant samples. IPA uses both experimentally verified and highly confident prediction data for the analysis. The significance levels were calculated using Fisher’s exact tests.

Gene ontology was performed using ArrayStar (DNASTAR). Grouping was performed on genes that had 2 folds difference between wildtype and hGFAP:CKO samples and have p-values less than 0.05.

### Real time qPCR

Total RNA was converted to cDNA using the SuperScript Kit (Life Technologies). For FACS-sorted cells with low concentrations, we used 5 μl of total RNA to convert into cDNA. *Gapdh* was used for normalizing the samples. Real time qPCR reactions were performed on ABI 3770 using SYBR Green Master Mix (Life Technologies). The significance levels were calculated using unpaired Student’s *t* test.

Dicer1Forward-ACCAGCGCTTAGAATTCCTGGGAG

Dicer1Reverse-GCTCAGAGTCCATTCCTTGC

*Dicer1* qPCR primers are located in exon 23 of *Dicer1* thus in the event of recombination, the amplicons will not be made.

GapdhF-TTGATGGCAACAATCTCCAC

GapdhR-CGTCCCGTAGACAAAATGGT

Aldh1l1F-CAGTAAACCTCCTGGCCAAA

Aldh1l1R-CCCTGTTTTCCCTACTTCCC

GfapF-TTTCTCGGATCTGGAGGTTG

GfapR-AGATCGCCACCTACAGGAAA

Aqp4F-TATCCAGTGGTTTGCCCAGT

Aqp4R-GCAATTGGACATTTGTTTGC

NesF- GAGTTCTCAGCCTCCAGCAG

NesR- AGATCGCTCAGATCCTGGAA

Fgfr3F-CTCCTGCTGGCTAGGTTCAG

Fgfr3R- GCCTGCGTGCTAGTGTTCT

GlastF- ATCTTCCCGGATGCCTTACT

GlastR- GCTTCTCATGAGGATGCTGC

### miRNA expression profiling

1.5 ng of total RNA was transcribed into cDNA using TaqMan Reverse Transcription Kit (Life Technologies) followed by an additional preamplication step using TaqMan PreAmp Kit (Life Technologies). Quantification of miRNAs was performed using the TaqMan Array Rodent MicroRNA A Card. Average Ct values below 31.05 were used as a cut-off point for miRNAs considered expressed. This average Ct value was chosen as a cut-off point as signals above this value is not detectable. These qPCR plates contain primers for U6 small RNA, which can be used to normalize the array signals. miRNA qPCR array data are available in the ArrayExpress database (www.ebi.ac.uk/arrayexpress) under accession number E-MTAB-3336.

## Results

### Aldh1l1-EGFP positive cells are dysregulated in hGFAP-Cre Dicer1/Dicer1 mutant forebrain

**Fig 1:**
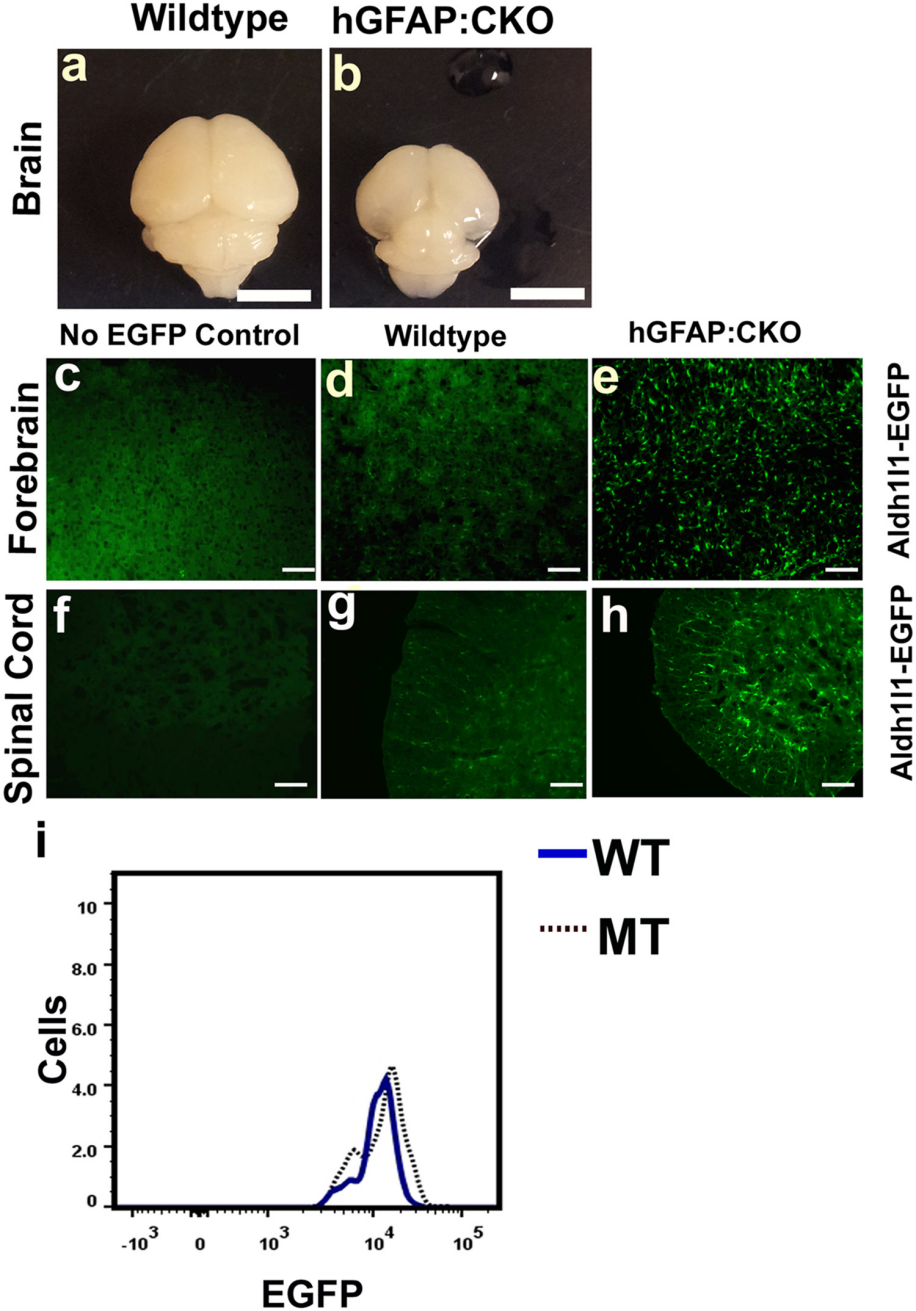
Analysis of hGFAP:CKO mice using the Aldh1l1-EGFP transgene shows presence of dysregulated astrocytes with increase EGFP signals. (a and b) P16 hGFAP:CKO forebrains have decreased sizes. (c,d, and e) P16 cortex and (f, g, and h) spinal cord sections indicate the presence of Aldh1l1-EGFP positive cells with higher EGFP signal in the hGFAP:CKO CNS. (i) The increase in EGFP signals can be visualized using FACS (blue represents P16 wildtype and dotted black represents P16 hGFAP:CKO Aldh1l1-EGFP positive cells). X axis in i represents EGFP levels in log2 scale. Y axis represents cell numbers. Scale bars in a and b represent 5 mm. Scale bars in c, d, and e represent 50 μm. Scale bars in f, g, and h represent 100 μm. FACS data for panel i is representative of 3 experiments. All histological experiments are representative data based on 3 independent observations.

Previous studies reported changes in astrocytes when ablation of *Dicer1* occurs before astrogliogenesis. Studies have found increased GFAP staining in these models while other found a decrease [3, 4]. Here, we used the Aldh1l1-EGFP transgene to further explore these astrocytes in models where miRNA ablation occurs before astrogliogenesis. In these reporter mice, EGFP proteins are activated by the *Aldh1l1* promoter, which resulted in the staining of astrocyte cell bodies. While some EGFP may localize to the processes of the astrocytes, the majority of the signals stay in the cell bodies. We combined Cre expressed under the human *GFAP* promoter with homozygous floxed *Dicer1* mice to generate astrocyte conditional knockout mice (hGFAP:CKO). The hGFAP-Cre transgene is activated in radial glial cells at E14.5 before the onset of astrogliogenesis in the forebrain [20]. Similar to other models where activation of Cre occurs in radial cells during neurogenesis, hGFAP:CKO mutant mice had smaller forebrains (Fig 1a and 1b) [3, 10, 11, 21, 22]. The mutant mice were ataxic and died between postnatal days 18 to 26. In these brains, a large number of Aldh1l1-EGFP positive cells were present in the forebrains and spinal cords of P16 mice (Fig 1c–1h, additional images of forebrain Aldh1l1-EGFP positive cells are shown in S1 Fig). This result indicates that astrogliogenesis can occur in the absence of mature miRNAs. Thus our data agreed with reports that found an increase in astrocytes when mature miRNAs were ablated during neurogenesis. However, while there were abundant Aldh1l1-EGFP cells, both histological data and FACS analysis indicated that hGFAP:CKO Aldh1l1-EGFP cells had increased EGFP signals in all regions of the central nervous system (Fig 1c–1i and S2 Fig).

We further assessed Aldh1l1-EGFP cells in the hGFAP:CKO mutant forebrains and found the presence of more Aldh1l1-EGFP positive cells in hGFAP:CKO mutant than in wildtype forebrains. Aldh1l1-EGFP cell numbers in hGFAP:CKO mutant forebrains (P16 wildtype (WT) = 122.2 ±19.2 n = 3, P16 hGFAP:CKO = 255.4±44.1 n = 3, p<0.05 per 500 μm^2^ unit, *t* test, unpaired, S3 Fig) were greater than in wildtype forebrains. In the cortex, astrocytes divide locally until around P7 [23]. We focused our investigation in P7 forebrains where astrocytes are still dividing to determine if proliferation was different. At this stage, hGFAP:CKO forebrain Aldh1l1-EGFP cells had increased proliferation compared to wildtype mice (13.6% ± 5% co-localization of ki67/EGFP for hGFAP:CKO vs 4% ± 1% ki67/EGFP for WT, n = 3 per genotype, p < 0.05, by *t*-test, unpaired, S4 Fig). As a reflection of this increased proliferation, more Aldh1l1-EGFP cells were also observed in the hGFAP:CKO forebrains (P7 WT = 141.5 ±19.8 n = 3, P7 hGFAP:CKO = 264.4 ±34.9, n = 3, p<0.05, per 500 μm^2^ unit, *t* test, unpaired, S5 Fig). FACS analysis also indicated an increase in the number of Aldh1l1-EGFP cells in mutant P7 forebrains (WT forebrains contained 19.7% ±1.1% Aldh1l1-EGFP positive cells and mutant forebrains contained 31.7% ± 3.5% Aldh1l1-EGFP positive cells, p<0.01, *t* test, unpaired, n = 3 per genotypes, S6 Fig).

### Evaluation of Aldh1l1-EGFP cell phenotype in mGFAP-Cre Dicer1/Dicer1 mutant forebrain

While the hGFAP:CKO mutants died prematurely and had smaller brains, animals appeared normal during development when we ablated the floxed *Dicer1* alleles with Cre expressed under the m*Gfap* promoter (mGFAP:CKO, line 77.6). These animals then developed ataxia and seizure at 6 weeks of age and also died prematurely at approximately 2 months [1]. In these lines, Cre is expressed early in postnatal life but after the occurrence of astrogliogenesis. This differs from human *GFAP* promoter, which is activated in radial glial cells during embryonic development and results in a deletion of *Dicer1* more exclusive to astrocytes.

**Fig 2:**
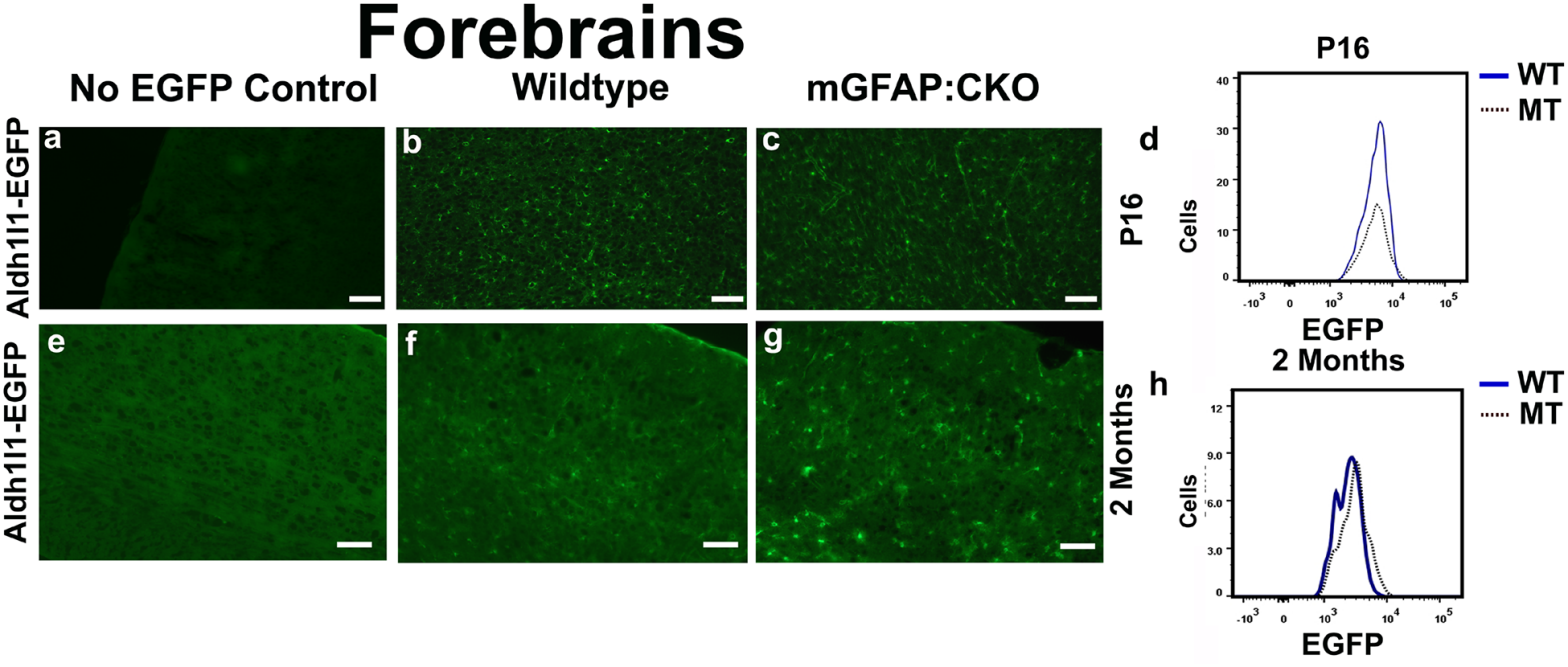
EGFP signals are increased in mGFAP:CKO forebrains. (a-b) Histological data and FACS analysis of P16 forebrains do not indicate a noticeable difference between wildtype and mGFAP:CKO animals (blue bar represents wildtype and dotted black line represents mGFAP:CKO forebrain in d). (e-g) While wildtype EGFP levels at 2 months are low and difficult to distinguish in the forebrains, mutant Aldh1l1-EGFP cells are bright and distinct. (h) FACS analysis also shows a dramatic increase in EGFP signal in 2 months old forebrain of mGFAP:CKO mutants (blue color represents wildtype, dotted black represents mGFAP:CKO). X axes in d and h represent EGFP levels in log2 scale. Y axes represent cell numbers at each EGFP levels. Scale bars in a, b, and c represent 100 μm. Scale bars in e, f, and g represent 50μm. FACS data for panel d are representative data based on 3 experiments. FACS data for 2 month old animals in panel h are representative data based on 2 different experiments. Histological experiments are representative data based on 3 independent observations.

Next we assessed these mutants to determine if Aldh1l1-EGFP positive cells also had up-regulated signals. Histological sections from mGFAP:CKO mutant did not exhibit noticeable up-regulation of EGFP signals in P16 forebrains (Fig 2a–2c). FACS analysis also did not show increased levels of fluorescence in Aldh1l1-EGFP positive cells (Fig 2d and S7 Fig). mGFAP:CKO mutants began to develop ataxia at 6 weeks and displayed astrogliosis which can be identified using GFAP antibodies (S8 Fig). In two-month old wildtype forebrains, EGFP signals were low and difficult to distinguish from background, whereas mutant cells had higher and more distinct EGFP signals (Fig 2e–2h and S9 Fig). Thus, while developing mGFAP:CKO animals did not have a distinct difference in forebrain Aldh1l1-EGFP signals, symptomatic mGFAP:CKO animals displayed up-regulated EGFP phenotype similar to hGFAP:CKO mutants.

### Aldh1l1-EGFP cells are disorganized in the cerebellums of developing mGFAP:CKO mice

**Fig 3:**
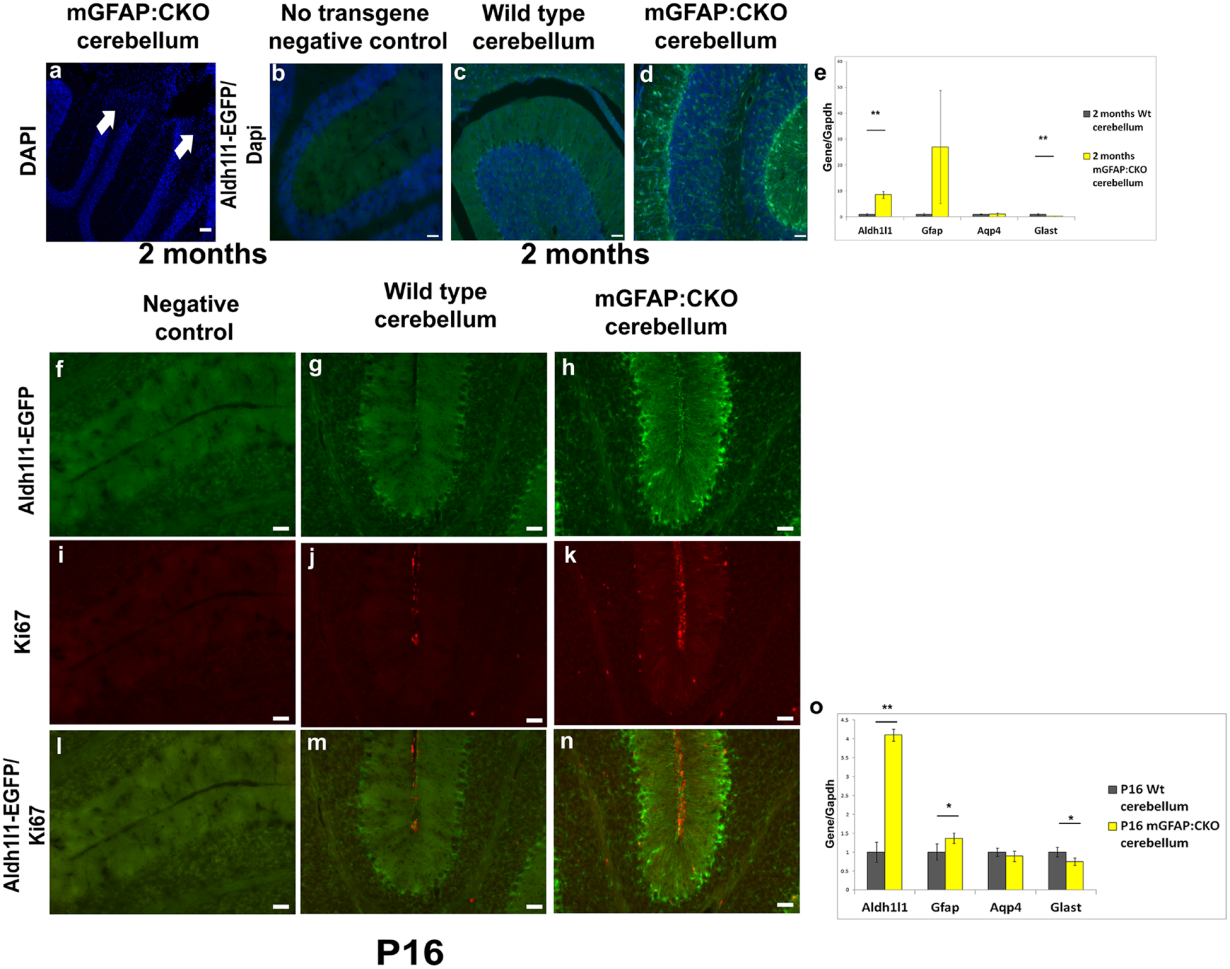
EGFP positive cells are disorganized and have increased EGFP signals in mGFAP:CKO cerebellums. (a) In symptomatic 2 month old mGFAP:CKO mice, massive cell loss was observed in mutant cerebellum (arrows indicate missing cells in cerebellar lobes). (b-d) EGFP positive cells had up-regulated signals and appeared in the molecular layers in the mutant cerebellums. (e) qRT-PCR also indicated up-regulated levels of *Aldh1l1* transcripts (p-value< 0.01, n = 3). (e) *Gfap* showed an upward trend suggesting astrogliosis (p-value = 0.05, n = 3). (e) *Aqp4* levels were similar between wildtype and mutant animals (p-value = 0.14, n = 3). (e) *Glast* level was decreased in mutant cerebellums (p-value < 0.01, n = 3). (f-h) In P16 mGFAP:CKO, defects with Aldh1l1-EGFP could already be observed in the cerebellum. Aldh1l1-EGFP cells were present in mutant molecular layer. (i-k) While ki67 positive cells were limited in wildtype cerebellum, we saw a noticeable increase in mutant cerebellum (ki67 cells are red). (l-m) These ki67 positive cells co-localized predominately with EGFP cells. *Aldh1l1* and *Gfap* transcripts were up-regulated while *Glast* had a small decrease expression level in mutant cerebellum. (o) No change was detected in *Aqp4* (*Aldh1l1* p-value< 0.01, *Aqp4* p-value = 0.16, *Gfap* p-value< 0.05, and *Glast* p-value< 0.05, n = 3). Scale bars in a-d and f-n represent 50 μm. Gray bars represent wildtype mice and yellow bars represent mGFAP:CKO mice in e and o. Asterisks in e and o indicate significant level (** represents p< 0.01 and * represents p< 0.05). Y axes in e and o represent the levels for gene of interest normalized to *Gapdh*. The average expression level of wildtype samples were normalized to 1. Unpaired t-test was used to determine p-values.

Although mGFAP:CKO mutant cerebellums appear to develop normally, neurodegeneration in the cerebellum occurs as these animals age. Histological examination of two-month-old symptomatic animals indicated cellular loss and disorganization of Aldh1l1-EGFP positive cells in the cerebellum (Fig 3a–3d). Additionally, real-time qPCR analysis of *Aldh1l1* levels indicated up-regulation in the mutant cerebellum (Fig 3e). During development, mGFAP:CKO mice did not exhibit ataxic behavior and gross features of their cerebellum appeared normal. However, we found abnormal localization of Aldh1l1-EGFP positive cells in P16 mGFAP:CKO cerebellums (Fig 3f–3h). While the molecular layers of wildtype cerebellums exhibited no clear Aldh1l1-EGFP cell bodies, mGFAP:CKO cerebellum molecular layers contained Aldh1l1-EGFP positive cells (Fig 3f–3h). EGFP signals also appeared elevated in the mutant Bergman glial cells (Fig 3f–3h), which is reflected in up-regulation of *Aldh1l1* transcript levels in the cerebellums of P16 mGFAP:CKO mice (Fig 3o). Additionally, we found small but significant up-regulation and down-regulation of *Gfap* and *Glast*, respectively (Fig 3o). Taken together, these data indicate that mGFAP:CKO cerebellums are dysregulated by P16.

Previous studies by Tao et. al. suggested that mGFAP:CKO cerebellum astrocytes are less differentiated [1]. We therefore assessed if Aldh1l1-EGFP positive cells have increased proliferation during development. Using ki67 antibodies as a marker for proliferation, we found that mGFAP:CKO cerebellums had increased number of ki67 positive cells throughout the molecular layer, while wildtype P16 cerebellum did not have any measurable dividing cells in the molecular layer (Fig 3i–3k). We found that 87.3% ± 1.5% of these ki67 positive cells in the molecular layer co-localized with Aldh1l1-EGFP positive cells (out of 27.5 ± 2.12 ki67 cells in molecular layer, n = 2 mutants) (Fig 3l–3n). Thus in the absence of mature miRNAs in mGFAP:CKO cerebellum, Aldh1l1-EGFP in the molecular layer are actively proliferating at P16.

### The expression profile of hGFAP:CKO Aldh1l1-EGFP cells displays immature and primary culture astrocyte phenotypes

**Fig 4:**
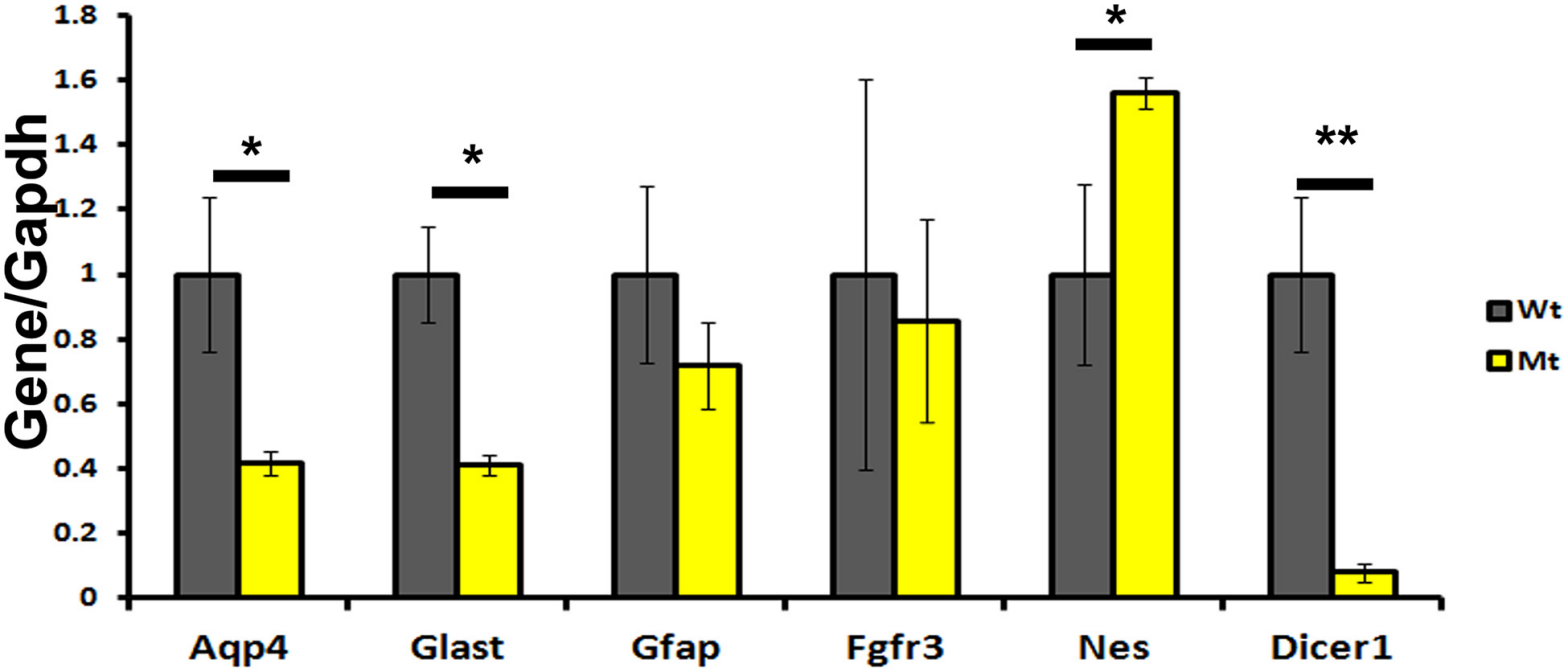
qRT-PCR indicates that FACS isolated hGFAP:CKO Aldh1l1-EGFP cells exhibit immature molecular phenotype. qRT-PCR indicated mutant cells have significantly down-regulated expression of *Aqp4* and *Glast (p-value< 0*.*05)*. The expression of *Gfap* did not reach significant down-regulation (p-value of *Gfap* is 0.09). *Fgfr3* levels appeared similar and *Nes* levels were up-regulated (p-value < 0.05). As expected, an amplicon that detected exon 23 of *Dicer1* had dramatically reduced levels in the mutant cells (p-values < 0.01). Unpaired t-test was used to determine p-values. Gray bars represent wildtype and yellow bars represent hGFAP:CKO. Asterisks indicate significant level (** represents p< 0.01 and * represents p< 0.05). Y axis represents expression levels for the gene of interest normalized to *Gapdh*. The average expression level of wildtype samples were normalized to 1. N = 3

The introduction of the Aldh1l1-EGFP transgene into these mice allowed us to isolate and analyze the molecular features of Aldh1l1-EGFP positive cells from young forebrains using FACS. Given the change in EGFP signals of the P7 hGFAP:CKO forebrains, we isolated Aldh1l1-EGFP positive cells from these mutants to perform further molecular characterization (S10 Fig). We obtained live cells that were 95–98% Aldh1l1-EGFP- positive after double sorting. Using qRT-PCR, we found down-regulation of exon 23 in *Dicer1* mRNA, which indicated that recombination occurred in these cells (Fig 4). Evaluation with qRT-PCR of known astrocyte genes in mutant cells showed that *Aqp4* and *Glast* were significantly down-regulated in mutant cells, suggesting that these cells were deficient in expressing genes involved in proper astrocyte functions (Fig 4, p-values for *Aqp4* and *Glast* <0.05, *t* test, unpaired). *Gfap* expression was not significantly down-regulated, which suggested that these cells already acquired astrocyte identity and were in the process of maturing (Fig 4, p-value for *Gfap* was 0.09, *t* test, unpaired). We also found *Fgfr3* transcript levels to be similar between hGFAP:CKO and wildtype cells, which suggests that acquisition of astrocyte identity occurred (Fig 4). *Nes*, a developmental marker, had a small significant increase, indicating immature phenotype in these mutant cells (Fig 4) [16, 24]. Overall, the down-regulation of *Aqp4* and up-regulation of *Nes* suggest that mutant Aldh1l1-EGFP cells exhibit an immature molecular signature [16].

To identify other features of the dysregulated Aldh1l1-EGFP cells, we performed microarray expression profiling of FACS isolated Aldh1l1-EGFP positive cells. Expression profiling was performed using RNA of Aldh1l1-EGFP positive cells isolated from P7 wildtype and hGFAP:CKO forebrains (n = 3 per genotype) and the Gene 1.0ST microarray chip. S1 Table contains the normalized expression data with corresponding p- values. In mutant cells, many genes associated with mature astrocytes were down-regulated and genes associated with immature astrocytes were up-regulated. For example, *Aqp4* and *Slc1a2* (-2.22, FDR = .08 and -2.12, FDR = .05, respectively, S1 Table) were down-regulated. Additionally genes associated with immature phenotypes such as *Nes* and *Vim* were up-regulated (1.78, FDR = .07 and 2.34, FDR = .05, respectively, S1 Table) [24]. We also analyzed the microarray data on genes involved in cell division to determine if they aligned with the histology data. Although we found that several genes involved in cell division such as Ccna1, Ccnb1, Ccnb2, and Cdkn1a had increased levels, their expression changes were not significant and did not indicate active proliferation. One possible explanation for this difference between the expression data and histological data may be that only a small subset of these mutant cells are proliferating. Our localization data with ki67 showed that only 13% of Aldh1l1-EGFP cells co-localized with this marker, thus the expression profile may not be sensitive enough to capture the change.

We used gene ontology to uncover additional features that were disrupted in hGFAP:CKO mutant Aldh1l1-EGFP positive cells. We found that differentially regulated genes (greater than or equal to 2-fold differences with p-value <0.05) in mutant Aldh1l1-EGFP positive cells fall into metabolic pathways such as cholesterol and lipid synthesis pathways (Table 1). While direct assessment of cholesterol level of the brain will need to be assessed, the change in expression of this of group of genes may indicate that the cholesterol levels of brain are altered [25].

**Table 1:** Pathway enriched in wildtype FACS cells compare to hGFAP:CKO FACS cells.

Gene Ontology Terms	P-Value
sterol biosynthetic process	4.27E-19
cholesterol biosynthetic process	2.61E-17
single-organism metabolic process	9.13E-17
sterol metabolic process	5.92E-15
alcohol biosynthetic process	8.17E-14
small molecule metabolic process	3.47E-13
cholesterol metabolic process	3.72E-13
organic hydroxy compound biosynthetic process	8.87E-13
steroid biosynthetic process	4.67E-12
alcohol metabolic process	4.45E-11
organic hydroxy compound metabolic process	5.61E-11
lipid metabolic process	1.99E-10
steroid metabolic process	2.03E-10
lipid biosynthetic process	2.06E-10
single-organism biosynthetic process	2.34E-09
small molecule biosynthetic process	3.95E-08
transport	6.54E-08
oxidation-reduction process	7.32E-08
establishment of localization	0.000000148
cellular lipid metabolic process	0.00000017
isoprenoid biosynthetic process	0.000000925
single-organism transport	0.00000494
generation of precursor metabolites and energy	0.00000919
fatty acid metabolic process	0.0000721
isoprenoid metabolic process	0.0000915
biological_process	0.0000929
sensory perception of smell	0.000117

We noticed that many of the down-regulated genes in hGFAP:CKO astrocytes were also down-regulated when we compared the FACS P7 wildtype cells to primary culture expression [16]. To investigate this further, we generated expression data of forebrain primary astrocytes from wildtype mice that were grown in culture for two weeks. Additionally, we sought to limit the heterogeneity by isolating Aldh1l1-EGFP positive cells in these cultures. At two weeks, primary astrocyte cultures had 50–75% Aldh1l1-EGFP positive cells (S11 Fig). When hGFAP:CKO cells and primary Aldh1l1-EGFP cells were compared to wildtype Aldh1l1-EGFP FACS cells, we found that hGFAP:CKO and primary culture Aldh1l1-EGFP cells shared many of the same differences from wildtype FACS isolated Aldh1l1-EGFP cells (Tables 2 and 3). 76% of the genes that were 2-fold down-regulated between hGFAP:CKO and wildtype FACS Aldh1l1-EGFP cells were also down-regulated in primary astrocytes (Table 2, column 2 and 4). On the other hand, 61% of the genes up-regulated in hGFAP:CKO Aldh1l1-EGFP cells were also up-regulated in the primary astrocyte cultures when compared to wildtype Aldh1l1-EGFP cells (Table 3, column 2 and 4). These data suggest that P7 hGFAP:CKO astrocytes acquired many of the molecular signatures of primary astrocyte cultures.

**Table 2:** Genes with >2-fold down-regulation in hGFAP:CKO Aldh1l1-EGFP that are also down-regulated in primary cultures.

Gene	Fold down- regulated in hGFAP:CKO	FDR	Fold changed in wildtype primary cells compared to wildtype FACS Aldh1l1-EGFP cells	FDR
Pla2g3	-7.77	0.039	-18.00	0.002
Gabrg1	-6.98	0.012	-25.99	0.003
Gpc5	-5.80	0.014	-14.54	0.001
Slc13a5	-5.34	0.010	-23.26	0.001
Mgll	-4.73	0.035	-3.72	0.019
Lcat	-3.82	0.048	-2.82	0.014
3110082D06Rik	-3.70	0.028	-2.88	0.031
Fmo1	-3.68	0.028	-13.77	0.003
Kcnj16	-3.65	0.014	-11.01	0.002
Hopx	-3.60	0.040	-3.63	0.014
Chst1	-3.38	0.014	-11.81	0.001
Grm5	-3.36	0.014	-9.64	0.001
Rarres1	-3.34	0.039	-1.65	0.085
Cyp7b1	-3.23	0.046	-5.06	0.009
Kcnip3	-3.22	0.038	-8.14	0.003
Il18	-3.22	0.012	-12.27	0.006
Cmtm5	-3.08	0.035	-12.89	0.004
Gjb6	-3.07	0.016	-5.25	0.001
Epas1	-3.00	0.039	-1.09	0.788
Eps8	-2.94	0.032	-3.63	0.006
Slc13a3	-2.90	0.043	-6.76	0.002
Baalc	-2.80	0.048	-2.74	0.013
Bex2	-2.74	0.039	-2.29	0.182
Idi1	-2.73	0.049	-1.73	0.449
Sc4mol	-2.61	0.038	-1.90	0.318
Rgs7	-2.58	0.023	-3.87	0.010
Tlr3	-2.53	0.028	-1.38	0.473
Ramp2	-2.52	0.023	-4.12	0.003
Luzp2	-2.47	0.037	-6.42	0.001
Thbs4	-2.35	0.043	-3.10	0.005
Dio2	-2.35	0.023	-11.05	0.001
Camk2g	-2.33	0.048	-2.10	0.023
Chrdl1	-2.31	0.035	-2.42	0.006
Lama4	-2.21	0.046	-2.09	0.023
Tmem218	-2.18	0.048	-1.51	0.328
Vldlr	-2.14	0.035	-4.22	0.010
Abr	-2.05	0.041	-3.57	0.003
Phactr3	-2.03	0.047	-4.28	0.002
Scn8a	-2.03	0.043	-5.04	0.001
Slc4a4	-2.01	0.048	-2.54	0.019

**Table 3:** Genes with >2-fold up-regulation in hGFAPCKO Aldh1l1-EGFP that are also up- regulated in primary cultures.

Gene	Fold up-regulated in hGFAP:CKO	FDR	Fold changed in wildtype primary cells compared to wildtype FACS Aldh1l1-EGFP cells	FDR
Cp	10.89	0.010	27.11	0.004
Pmepa1	5.47	0.010	2.26	0.462
Fstl1	5.42	0.027	4.48	0.005
Tagln2	5.18	0.042	9.64	0.004
Fn1	5.07	0.012	28.72	0.002
Sfp4	4.17	0.010	1.12	0.613
Susd2	4.06	0.050	1.42	0.295
Igf2r	3.74	0.040	1.96	0.075
Adam12	3.65	0.019	4.80	0.028
Elmo1	3.61	0.014	1.57	0.079
Etl4	3.46	0.039	2.06	0.037
Frzb	3.43	0.014	1.03	0.944
Angptl2	3.42	0.024	2.21	0.029
Pcgf2	3.35	0.039	1.29	0.274
Tmem123	3.34	0.040	3.57	0.008
Grb10	3.20	0.041	1.41	0.183
Galnt7	3.15	0.016	1.19	0.449
Anxa2	2.77	0.037	6.74	0.002
Itga1	2.71	0.035	8.81	0.001
Tmem2	2.68	0.038	1.80	0.058
Sorl1	2.58	0.041	1.58	0.092
Cpd	2.56	0.045	2.12	0.022
Man2a1	2.54	0.036	1.99	0.043
Prr11	2.50	0.031	1.35	0.274
Pgpep1	2.50	0.035	1.20	0.427
Tns1	2.48	0.038	2.11	0.014
Fbn1	2.46	0.035	3.41	0.004
Lama5	2.44	0.035	4.04	0.002
Prtg	2.43	0.042	2.51	0.024
Nox4	2.42	0.048	2.46	0.017
Ltbp2	2.40	0.035	5.39	0.003
Clcn5	2.39	0.031	1.17	0.589
Slc25a24	2.37	0.047	2.07	0.019
Kif4	2.32	0.035	2.40	0.006
Xpo1	2.29	0.040	1.06	0.821
Plod2	2.22	0.049	2.12	0.208
Socs2	2.21	0.047	2.75	0.021
Il1rap	2.18	0.042	2.62	0.019
Bdkrb2	2.18	0.032	3.69	0.090
Pmch	2.15	0.045	1.69	0.045
Klf5	2.07	0.042	2.85	0.022
Jazf1	2.05	0.039	3.14	0.008

### Identification of miRNAs expressed in P7 Aldh1l1-EGFP cells and their potential targets

To better understand how miRNAs may regulate astrocyte development and function, we identified the miRNAs that were expressed in P7 forebrain Aldh1l1-EGFP cells using Taqman Low Density Arrays. Additionally, we used Ingenuity Pathway Analysis (IPA) software to evaluate potential upstream regulator of the microarray data. Using the Taqman Low Density Arrays, we found that Aldh1l1-EGFP cells expressed many miRNAs (S2 Table). Several of the miRNAs on the list from P7 wildtype forebrain astrocytes such as *miR-21*, *miR-223*, *miR-146a* and *miR-181* (S2 Table) have been previously shown to regulate astrocyte functions [26–30]. However, the roles of most of these miRNAs in astrocytes are not known. We used IPA to determine whether genes with increased expression levels in hGFAP:CKO Aldh1l1-EGFP cells are targets of these miRNAs (S2 Table). Given that the effect of miRNAs on a gene may be subtle, we included all up-regulated genes for this analysis. As a negative control for this analysis, we also performed this IPA analysis on down-regulated genes. IPA identified various upstream regulators for many of these genes including miRNAs. While IPA identified several miRNAs expressed in astrocytes with the up-regulated set of genes, the data set only predicted two miRNAs with the down-regulated genes (S3 Table). The potential targets of miRNAs expressed by Aldh1l1-EGFP positive cells are listed in Table 4 (miRNAs predicted to be upstream regulator but did not have expression in Aldh1l1-EGFP cells are not shown on the list). While the relationship of these miRNAs and their potential targets will need to be further evaluated, the IPA analysis was able to identify two miRNAs, miR-181a and miR-125b that have been previously reported to regulate astrocytes [28, 31, 32]. Interestingly, we found that many of the genes are shared potential targets of different miRNAs in this analysis (Table 4), which suggests that different Aldh1l1-EGFP miRNAs may have redundant targets to ensure that a particular gene is down-regulated. Further experimental analysis of these miRNAs individually will be necessary to determine if they share similar targets and to determine the mechanism in which they regulate astrocyte maturation and functions.

**Table 4:** IPA predicted miRNAs from analysis of up-regulated genes that have expression in Aldh1l1-EGFP cells.

MiRNAs	p-value of overlap	Target molecules in dataset
miR-181a-5p	2.54E-04	BCLAF1,CREB1,DDX5,ENPP1,H3F3A/H3F3B,HMGB2,HOXC8,LBR,LMO1,MB21D2,PBX3,PCGF2,PRTG,RAD21,SCN9A,SHOC2,SSX2IP,TGFBR1,TNS1,TRANK1
miR-101-3p	2.86E-03	CREB1,EIF4G2,EZH2,FEM1C,GALNT7,JAK2,KIAA1217,PBX3,PDE4D,RAB14,SSX2IP,STMN1,TGFBR1,ZBTB18
miR-381-3p	4.74E-03	CREB1,EIF4G2,EXO1,GALNT7,HMGB1,HMGB2,HOXC8,KIAA1217,LIN9,PATL1,TGFBR1,TMEM260,XPO1,ZBTB18
let-7	8.35E-03	CDCA2,EZH2,MCM3,MCM5
miR-26a-5p	1.18E-02	CDKN1C,CILP,CREB1,EIF4G2,EZH2,FAM118A,GALNT7,PDE4D,PRTG,SSX2IP,TMEM194A,TMEM260,ZBTB18
miR-27a-3p	1.62E-02	BCLAF1,CA12,CREB1,ELMO1,ENPP1,GALNT7,GDF6,H3F3A/H3F3B,PRTG,RAB14,RET,SMCHD1,SSX2IP,TGFBR1,XPO1,ZBTB18
miR-425-5p	2.91E-02	BCLAF1,CREB1,DUSP16,SSX2IP,TMEM260
miR-204-5p	3.49E-02	CREB1,DUSP16,ELMO1,HOXC8,JAK2,PRR11,PRTG,RAB14,TGFBR1,TMEM194A
miR-125b-5p	4.66E-02	ANGPT2,CREB1,ENPP1,FAM118A,GALNT7,H3F3A/H3F3B,KIAA0317,PPAT,PRTG,RET,ST6GAL1,TMEM194A
miR-197-3p	4.83E-02	CILP,FRZB,PGPEP1,RAD51B,ZBTB18

## Discussion

While other studies mainly used GFAP antibodies to understand the role of miRNAs in astrocyte development, here we used a pan-astrocyte marker, Aldh1l1-EGFP, to characterize novel changes of astrocytes in two mouse *Dicer1* models. Our study found that the ablation of *Dicer1* with hGFAP-Cre or mGFAP-Cre produced different phenotypes in astrocytes as assessed from the features of Aldh1l1-EGFP cells.

In this study, we found that Aldh1l1-EGFP positive cells in young hGFAP:CKO forebrains displayed up-regulated EGFP signals and molecular phenotypes of immature and primary astrocytes, while Aldh1l1-EGFP positive cells appeared normal in young mGFAP:CKO forebrains. One possible reason for this difference may be that miRNAs affect astrocytes differently during different developmental time points similar to how neurons are affected differently depending on the timing of the ablation of *Dicer1* [8]. Cre is activated prior to astrogliogenesis in the hGFAP:CKO line, whereas it is expressed after astrogliogenesis in the mGFAP:CKO line. Another reason may be that the hGFAP:CKO astrocytes are in a reactive state since brain malformations occur in the hGFAP:CKO line. *Aldh1l1* is known to be up-regulated in reactive astrocytes [14]. mGFAP:CKO Aldh1l1-EGFP cells also had a more dramatic increase in EGFP signals during symptomatic disease state, further suggesting that the reactive state of astrocytes may be contributing to the phenotype that we see in the hGFAP:CKO mutants. The molecular profile that we obtained from hGFAP:CKO Aldh1l1-EGFP cells contained some genes that have been implicated in the profile of reactive astrocytes [33]. For example, one of the most highly expressed genes in the reactive astrocyte data was *Cp*, which was also highly up-regulated in the hGFAP:CKO mutant samples. However, other highly expressed genes in reactive astrocytes such as *Lcn* did not appear in our database. *Gfap* was also not up-regulated in hGFAP:CKO mutant animals. Reactive astrocytes are now known to be heterogeneous depending on the context of the brain injury [33, 34]. Therefore the possibility that the hGFAP:CKO Aldh1l1-EGFP cells are a type of previously unknown reactive astrocyte cannot be ruled out. Thus the phenotype seen in hGFAP:CKO Aldh1l1-EGFP cells may be the result of both the cells being in a reactive state and the loss of mature miRNAs.

Using the Aldh1l1-EGFP transgene, we did not notice gross changes of astrocytes in the mGFAP:CKO forebrain at P16. The Aldh1l1-EGFP transgene did allow us to observe changes to Aldh1l1-EGFP cells in mGFAP:CKO cerebellum at P16. Previous study did not notice differences in P15 mGFAP:CKO cerebellum. We found that Aldh1l1-EGFP cells were localized in the molecular layer of mutant P16 cerebellum while wildtype Aldh1l1-EGFP cells were not. Additionally, these Aldh1l1-EGFP cells in the molecular layers were proliferating. At this stage, we also found small but significant up-regulation of *Gfap*. Thus our data indicate that dysregulated features are present in P16 mGFAP:CKO mice.

Our genomic approach allowed us to further characterize astrocytes in hGFAP:CKO mice as well as identifying several interesting features. One interesting aspect was that the cholesterol gene pathway was down-regulated in mutant cells. While additional research will be required to assess if cholesterol levels are affected in mutant brains, this gene expression signature is potentially interesting for future studies since cholesterols from astrocytes have been shown to be involved in the formation of synapse in neurons [25].

Currently, the *in vivo* relevance of primary astrocytes is not well understood, although it has been hypothesized that primary cells may be more similar to reactive astrocytes [16, 33]. Here we were able to discover another situation where *in vivo* cells resembled many of the characteristics of primary astrocytes.

In conclusion, our data provide new insights into the phenotypes of astrocytes in mouse models where *Dicer1* is deleted with different astrocyte Cre lines. We used the Aldh1l1-EGFP transgene to describe previously unknown aspects of astrocytes in these mutants. Additionally, this study showed that Aldh1l1-EGFP transgene can be a useful marker in understanding asymptomatic developing phases of mGFAP:CKO neurodegenerative disease model and potentially in understanding the roles of developmental astrocytes in other neurodegenerative diseases.

## Supporting Information

S1 Fig Image of Aldh1l1-EGFP cells in P16 wildtype and hGFAP:CKO forebrains. Scale bars represent to 50μm.
(TIF)

S2 Fig Gating strategies for P16 wildtype and hGFAP:CKO forebrains. (TIF)

S3 Fig Number of Aldh1l1-EGFP cells in P16 wildtype and hGFAP:CKO forebrains. Long horizontal lines represent mean and short horizontal lines represent standard deviation.
(TIF)

S4 Fig Percent co-localization of ki67 and Aldh1l1-EGFP cells in P7 wildtype and hGFAP:CKO forebrains. Long horizontal lines represent mean and short horizontal lines represent standard deviation.
(TIF)

S5 Fig Number of Aldh1l1-EGFP cells in P7 wildtype and hGFAP:CKO forebrains. Long horizontal lines represent mean and short horizontal lines represent standard deviation.
(TIF)

S6 Fig Percent of Aldh1l1-EGFP cells in P7 wildtype and hGFAP:CKO forebrains using FACS analysis. Long horizontal lines represent mean and short horizontal lines represent standard deviation.
(TIF)

S7 Fig Gating strategies for P16 wildtype and mGFAP:CKO forebrains. (TIF)

S8 Fig GFAP staining is increased in two months old mGFAP:CKO mutants. Scale bars representing 100μm.
(TIF)

S9 Fig Gating strategies for 2 months old wildtype and mGFAP:CKO forebrains. (TIF)

S10 Fig Gating strategies for P7 wildtype and hGFAP:CKO forebrains. (TIF)

S11 Fig Gating strategies for primary Aldh1l1-EGFP positive astrocytes. (TIF)

S1 Table Normalized microarray expression data of Aldh1l1-EGFP positive cells. (XLSX)

S2 Table miRNA qPCR array data. (XLSX)

S3 Table Predicted upstream miRNA using genes downregulated in microarray. (XLSX)

## References

[1] Tao J, Wu H, Lin Q, Wei W, Lu XH, Cantle JP, et al Deletion of astroglial Dicer causes non-cell-autonomous neuronal dysfunction and degeneration. J Neurosci. 2011;31(22):8306–19. Epub 2011/06/03. doi: 31/22/8306 [pii] 10.1523/JNEUROSCI.0567-11.2011 . 21632951 PMC3500097

[2] Andersson T, Rahman S, Sansom SN, Alsio JM, Kaneda M, Smith J, et al Reversible block of mouse neural stem cell differentiation in the absence of dicer and microRNAs. PLoS One. 2010;5(10):e13453 Epub 2010/10/27. 10.1371/journal.pone.0013453 . 20976144 PMC2956652

[3] McLoughlin HS, Fineberg SK, Ghosh LL, Tecedor L, Davidson BL. Dicer is required for proliferation, viability, migration and differentiation in corticoneurogenesis. Neuroscience. 2012;223:285–95. Epub 2012/08/18. doi: S0306-4522(12)00829-9 [pii] 10.1016/j.neuroscience.2012.08.009 . 22898830 PMC3472421

[4] Zheng K, Li H, Zhu Y, Zhu Q, Qiu M. MicroRNAs are essential for the developmental switch from neurogenesis to gliogenesis in the developing spinal cord. J Neurosci. 2010;30(24):8245–50. Epub 2010/06/18. doi: 30/24/8245 [pii] 10.1523/JNEUROSCI.1169-10.2010 . 20554876 PMC2918643

[5] Volvert ML, Rogister F, Moonen G, Malgrange B, Nguyen L. MicroRNAs tune cerebral cortical neurogenesis. Cell Death Differ. 2012;19(10):1573–81. Epub 2012/08/04. doi: cdd201296 [pii] 10.1038/cdd.2012.96 . 22858543 PMC3438503

[6] Zheng K, Li H, Huang H, Qiu M. MicroRNAs and glial cell development. Neuroscientist. 2011;18(2):114–8. Epub 2011/05/11. doi: 1073858411398322 [pii] 10.1177/1073858411398322 . 21555783 PMC3762876

[7] Kuang Y, Liu Q, Shu X, Zhang C, Huang N, Li J, et al Dicer1 and MiR-9 are required for proper Notch1 signaling and the Bergmann glial phenotype in the developing mouse cerebellum. Glia. 2012;60(11):1734–46. Epub 2012/07/28. 10.1002/glia.22392 . 22836445

[8] Li Q, Bian S, Hong J, Kawase-Koga Y, Zhu E, Zheng Y, et al Timing specific requirement of microRNA function is essential for embryonic and postnatal hippocampal development. PLoS One. 2011;6(10):e26000 Epub 2011/10/13. 10.1371/journal.pone.0026000 PONE-D-11-09223 [pii]. . 21991391 PMC3186801

[9] Saurat N, Andersson T, Vasistha NA, Molnar Z, Livesey FJ. Dicer is required for neural stem cell multipotency and lineage progression during cerebral cortex development. Neural Dev. 2013;8:14. Epub 2013/07/31. doi: 1749-8104-8-14 [pii] 10.1186/1749-8104-8-14 . 23895693 PMC3737057

[10] Kawase-Koga Y, Otaegi G, Sun T. Different timings of Dicer deletion affect neurogenesis and gliogenesis in the developing mouse central nervous system. Dev Dyn. 2009;238(11):2800–12. Epub 2009/10/07. 10.1002/dvdy.22109 . 19806666 PMC2831750

[11] De Pietri Tonelli D, Pulvers JN, Haffner C, Murchison EP, Hannon GJ, Huttner WB. miRNAs are essential for survival and differentiation of newborn neurons but not for expansion of neural progenitors during early neurogenesis in the mouse embryonic neocortex. Development. 2008;135(23):3911–21. Epub 2008/11/11. doi: 135/23/3911 [pii] 10.1242/dev.025080 . 18997113 PMC2798592

[12] Nishino J, Kim I, Chada K, Morrison SJ. Hmga2 promotes neural stem cell self-renewal in young but not old mice by reducing p16Ink4a and p19Arf Expression. Cell. 2008;135(2):227–39. Epub 2008/10/30. doi: S0092-8674(08)01139-2 [pii] 10.1016/j.cell.2008.09.017 . 18957199 PMC2582221

[13] Tien AC, Tsai HH, Molofsky AV, McMahon M, Foo LC, Kaul A, et al Regulated temporal-spatial astrocyte precursor cell proliferation involves BRAF signalling in mammalian spinal cord. Development. 2012;139(14):2477–87. Epub 2012/06/08. doi: dev.077214 [pii] 10.1242/dev.077214 . 22675209 PMC3383225

[14] Yang Y, Vidensky S, Jin L, Jie C, Lorenzini I, Frankl M, et al Molecular comparison of GLT1+ and ALDH1L1+ astrocytes in vivo in astroglial reporter mice. Glia. 2010;59(2):200–7. Epub 2010/11/04. 10.1002/glia.21089 . 21046559 PMC3199134

[15] Harfe BD, McManus MT, Mansfield JH, Hornstein E, Tabin CJ. The RNaseIII enzyme Dicer is required for morphogenesis but not patterning of the vertebrate limb. Proc Natl Acad Sci U S A. 2005;102(31):10898–903. Epub 2005/07/26. doi: 0504834102 [pii] 10.1073/pnas.0504834102 . 16040801 PMC1182454

[16] Cahoy JD, Emery B, Kaushal A, Foo LC, Zamanian JL, Christopherson KS, et al A transcriptome database for astrocytes, neurons, and oligodendrocytes: a new resource for understanding brain development and function. J Neurosci. 2008;28(1):264–78. Epub 2008/01/04. doi: 28/1/264 [pii] 10.1523/JNEUROSCI.4178-07.2008 . 18171944 PMC6671143

[17] Molofsky AV, Glasgow SM, Chaboub LS, Tsai HH, Murnen AT, Kelley KW, et al Expression profiling of Aldh1l1-precursors in the developing spinal cord reveals glial lineage-specific genes and direct Sox9-Nfe2l1 interactions. Glia. 2013;61(9):1518–32. Epub 2013/07/11. 10.1002/glia.22538 . 23840004 PMC3909648

[18] Smyth GK. Linear models and empirical bayes methods for assessing differential expression in microarray experiments. Stat Appl Genet Mol Biol. 2004;3:Article3 Epub 2006/05/02. 10.2202/1544-6115.1027 . 16646809

[19] Benjamini Y, Hochberg Y. Controlling the false discovery rate: a practical and powerful approach to multiple testing. Journal of the Royal Statistical Society Series B (Methodological). 1995;57(1):289–300.

[20] Anthony TE, Heintz N. Genetic lineage tracing defines distinct neurogenic and gliogenic stages of ventral telencephalic radial glial development. Neural Dev. 2008;3:30. Epub 2008/11/07. doi: 1749-8104-3-30 [pii] 10.1186/1749-8104-3-30 . 18986511 PMC2637863

[21] Kuang Y, Liu Q, Shu X, Zhang C, Huang N, Li J, et al Dicer1 and MiR-9 are required for proper Notch1 signaling and the Bergmann glial phenotype in the developing mouse cerebellum. Glia. 60(11):1734–46. Epub 2012/07/28. 10.1002/glia.22392 . 22836445

[22] Nigro A, Menon R, Bergamaschi A, Clovis YM, Baldi A, Ehrmann M, et al MiR-30e and miR-181d control radial glia cell proliferation via HtrA1 modulation. Cell Death Dis. 2012;3:e360. Epub 2012/08/03. doi: cddis201298 [pii] 10.1038/cddis.2012.98 . 22854828 PMC3434671

[23] Tsai HH, Li H, Fuentealba LC, Molofsky AV, Taveira-Marques R, Zhuang H, et al Regional astrocyte allocation regulates CNS synaptogenesis and repair. Science. 2012;337(6092):358–62. Epub 2012/06/30. doi: science.1222381 [pii] 10.1126/science.1222381 . 22745251 PMC4059181

[24] Laywell ED, Rakic P, Kukekov VG, Holland EC, Steindler DA. Identification of a multipotent astrocytic stem cell in the immature and adult mouse brain. Proc Natl Acad Sci U S A. 2000;97(25):13883–8. Epub 2000/11/30. doi: 10.1073/pnas.250471697 250471697 [pii]. . 11095732 PMC17670

[25] Pfrieger FW, Ungerer N. Cholesterol metabolism in neurons and astrocytes. Prog Lipid Res. 2011;50(4):357–71. Epub 2011/07/12. doi: S0163-7827(11)00031-2 [pii] 10.1016/j.plipres.2011.06.002 . 21741992

[26] Glasgow SM, Laug D, Brawley VS, Zhang Z, Corder A, Yin Z, et al The miR-223/nuclear factor I-A axis regulates glial precursor proliferation and tumorigenesis in the CNS. J Neurosci. 2013;33(33):13560–8. Epub 2013/08/16. doi: 33/33/13560 [pii] 10.1523/JNEUROSCI.0321-13.2013 . 23946414 PMC3742938

[27] Jovicic A, Roshan R, Moisoi N, Pradervand S, Moser R, Pillai B, et al Comprehensive expression analyses of neural cell-type-specific miRNAs identify new determinants of the specification and maintenance of neuronal phenotypes. J Neurosci. 2013;33(12):5127–37. Epub 2013/03/22. doi: 33/12/5127 [pii] 10.1523/JNEUROSCI.0600-12.2013 . 23516279 PMC6705001

[28] Hutchison ER, Kawamoto EM, Taub DD, Lal A, Abdelmohsen K, Zhang Y, et al Evidence for miR-181 involvement in neuroinflammatory responses of astrocytes. Glia. 2013;61(7):1018–28. Epub 2013/05/08. 10.1002/glia.22483 . 23650073 PMC4624280

[29] Iyer A, Zurolo E, Prabowo A, Fluiter K, Spliet WG, van Rijen PC, et al MicroRNA-146a: a key regulator of astrocyte-mediated inflammatory response. PLoS One. 2012;7(9):e44789. Epub 2012/10/03. doi: 10.1371/journal.pone.0044789 PONE-D-12-15409 [pii]. . 23028621 PMC3441440

[30] Bhalala OG, Pan L, Sahni V, McGuire TL, Gruner K, Tourtellotte WG, et al microRNA-21 regulates astrocytic response following spinal cord injury. J Neurosci. 2012;32(50):17935–47. Epub 2012/12/15. doi: 32/50/17935 [pii] 10.1523/JNEUROSCI.3860-12.2012 . 23238710 PMC3538038

[31] Ouyang YB, Lu Y, Yue S, Giffard RG. miR-181 targets multiple Bcl-2 family members and influences apoptosis and mitochondrial function in astrocytes. Mitochondrion. 2011;12(2):213–9. Epub 2011/10/01. doi: S1567-7249(11)00282-0 [pii] 10.1016/j.mito.2011.09.001 . 21958558 PMC3250561

[32] Pogue AI, Cui JG, Li YY, Zhao Y, Culicchia F, Lukiw WJ. Micro RNA-125b (miRNA-125b) function in astrogliosis and glial cell proliferation. Neurosci Lett. 476(1):18–22. Epub 2010/03/30. doi: S0304-3940(10)00363-0 [pii] 10.1016/j.neulet.2010.03.054 . 20347935

[33] Zamanian JL, Xu L, Foo LC, Nouri N, Zhou L, Giffard RG, et al Genomic analysis of reactive astrogliosis. J Neurosci. 2012;32(18):6391–410. Epub 2012/05/04. doi: 32/18/6391 [pii] 10.1523/JNEUROSCI.6221-11.2012 . 22553043 PMC3480225

[34] Sirko S, Behrendt G, Johansson PA, Tripathi P, Costa M, Bek S, et al Reactive glia in the injured brain acquire stem cell properties in response to sonic hedgehog. [corrected]. Cell Stem Cell. 2013;12(4):426–39. Epub 2013/04/09. doi: S1934-5909(13)00095-7 [pii] 10.1016/j.stem.2013.01.019 . 23561443

