# Supplementary figures and images for: Understanding the Role of Dicer in Astrocyte Development
Source: PLoS One. 2015 May 11;10(5):e0126667. doi: 10.1371/journal.pone.0126667 (PMC4427179; doi:10.1371/journal.pone.0126667)

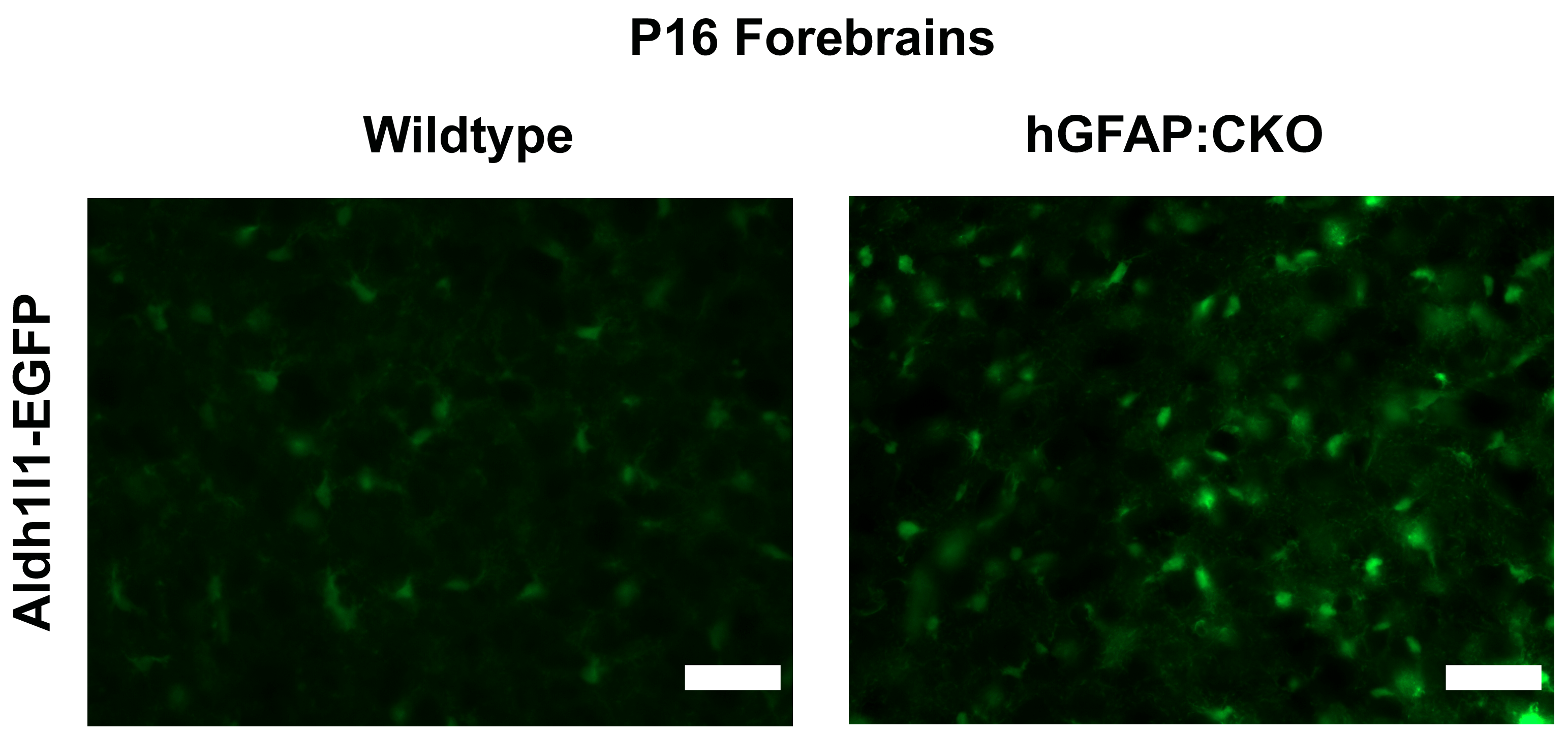

Supplement: S1 Fig — Scale bars represent to 50μm. (TIF) [file pone.0126667.s001.tif]

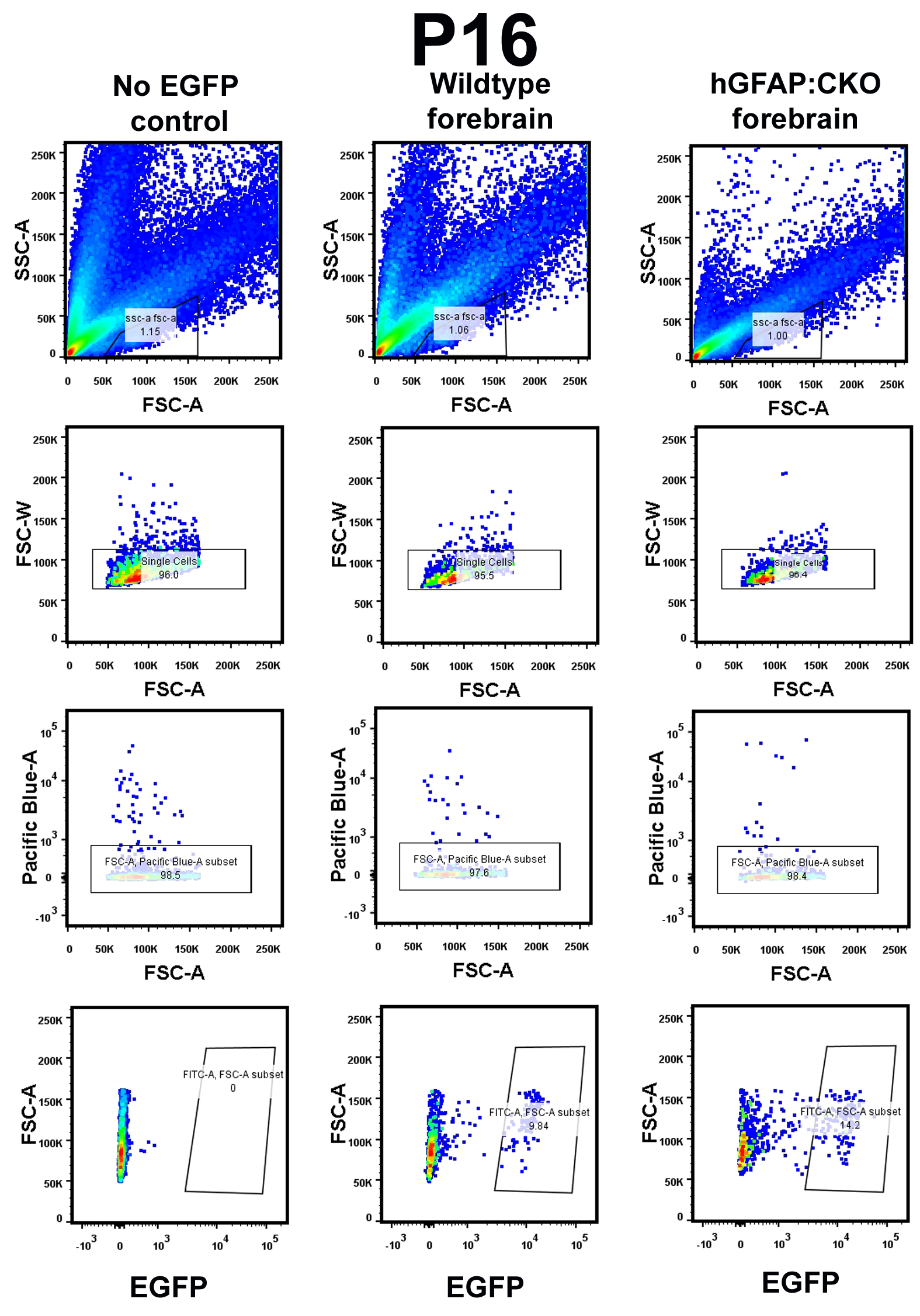

Supplement: S2 Fig — (TIF) [file pone.0126667.s002.tif]

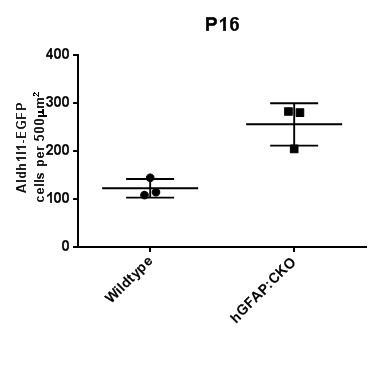

Supplement: S3 Fig — Long horizontal lines represent mean and short horizontal lines represent standard deviation. (TIF) [file pone.0126667.s003.tif]

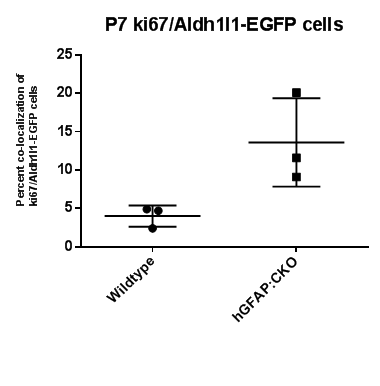

Supplement: S4 Fig — Long horizontal lines represent mean and short horizontal lines represent standard deviation. (TIF) [file pone.0126667.s004.tif]

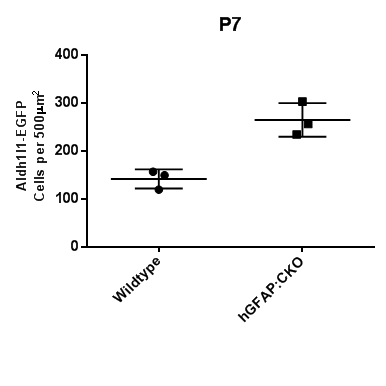

Supplement: S5 Fig — Long horizontal lines represent mean and short horizontal lines represent standard deviation. (TIF) [file pone.0126667.s005.tif]

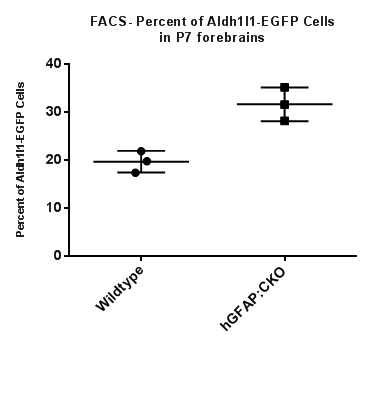

Supplement: S6 Fig — Long horizontal lines represent mean and short horizontal lines represent standard deviation. (TIF) [file pone.0126667.s006.tif]

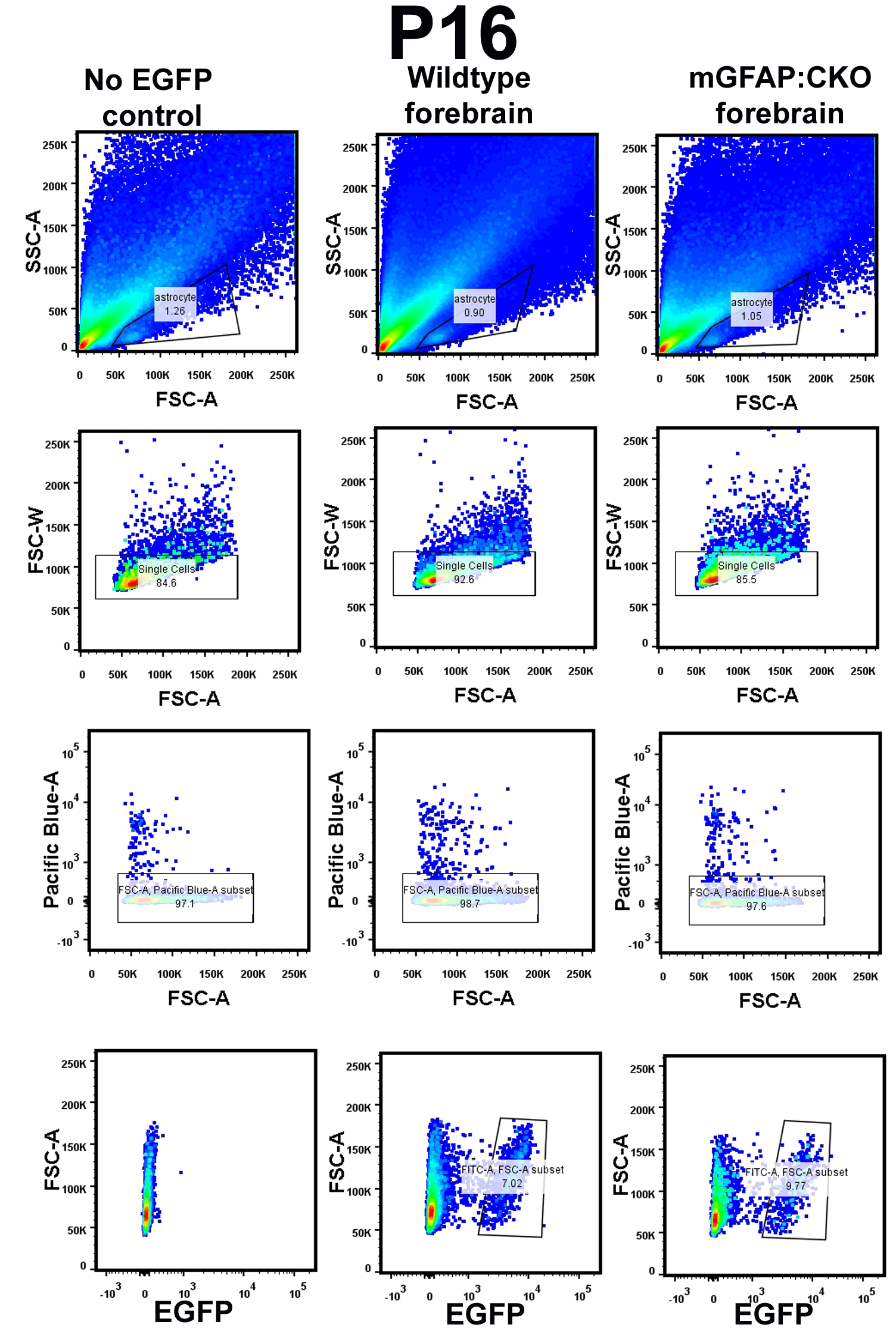

Supplement: S7 Fig — (TIF) [file pone.0126667.s007.tif]

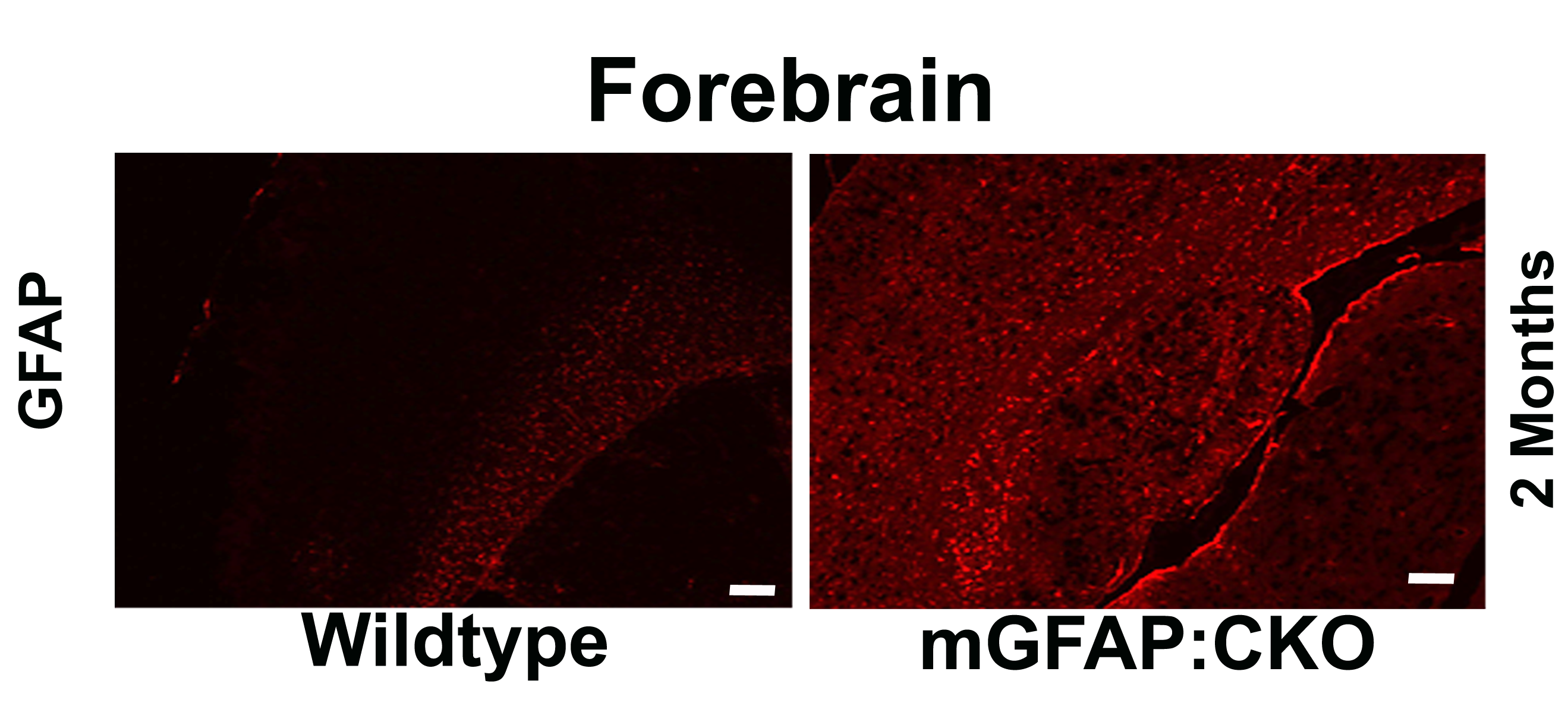

Supplement: S8 Fig — Scale bars representing 100μm. (TIF) [file pone.0126667.s008.tif]

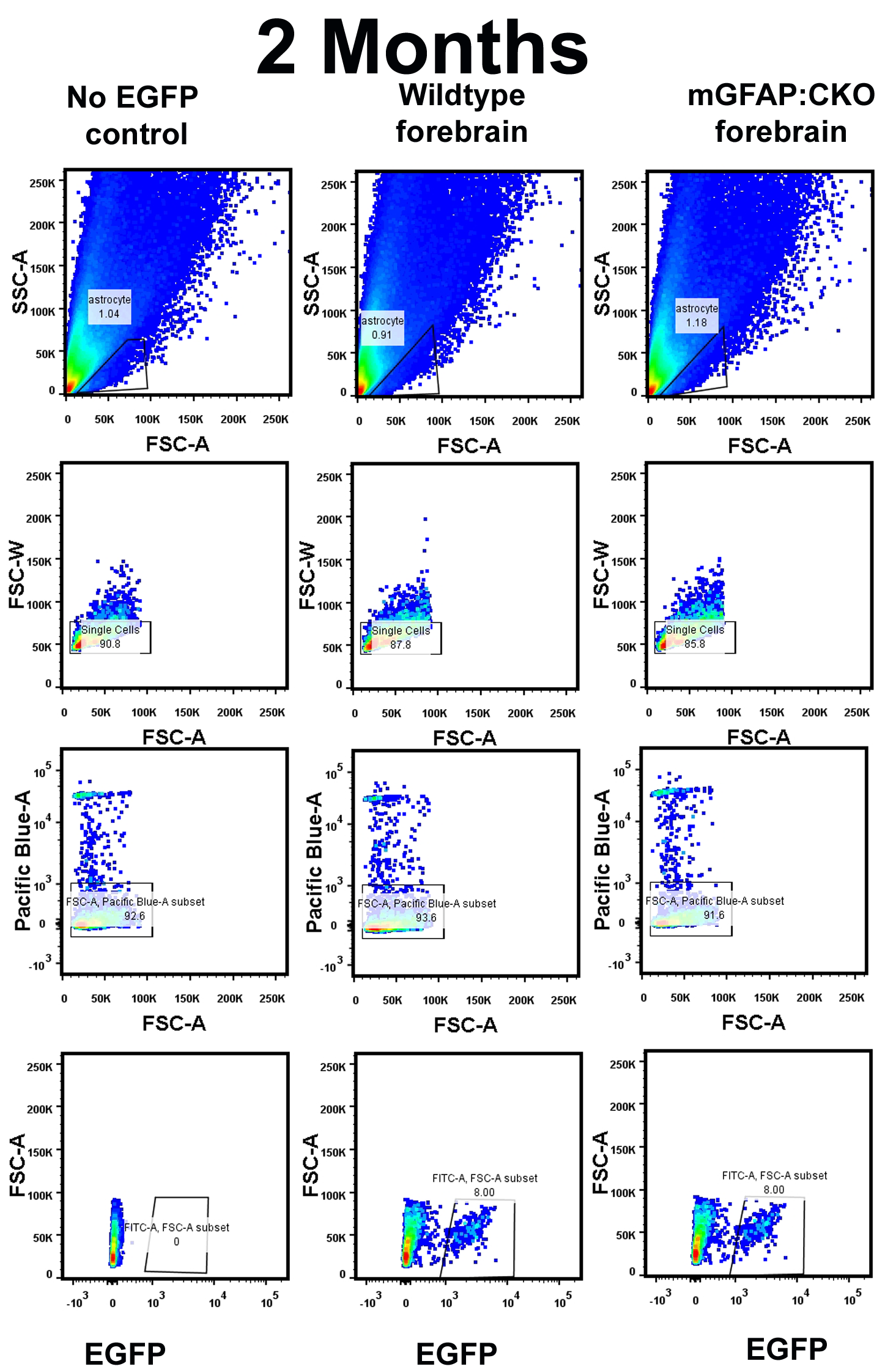

Supplement: S9 Fig — (TIF) [file pone.0126667.s009.tif]

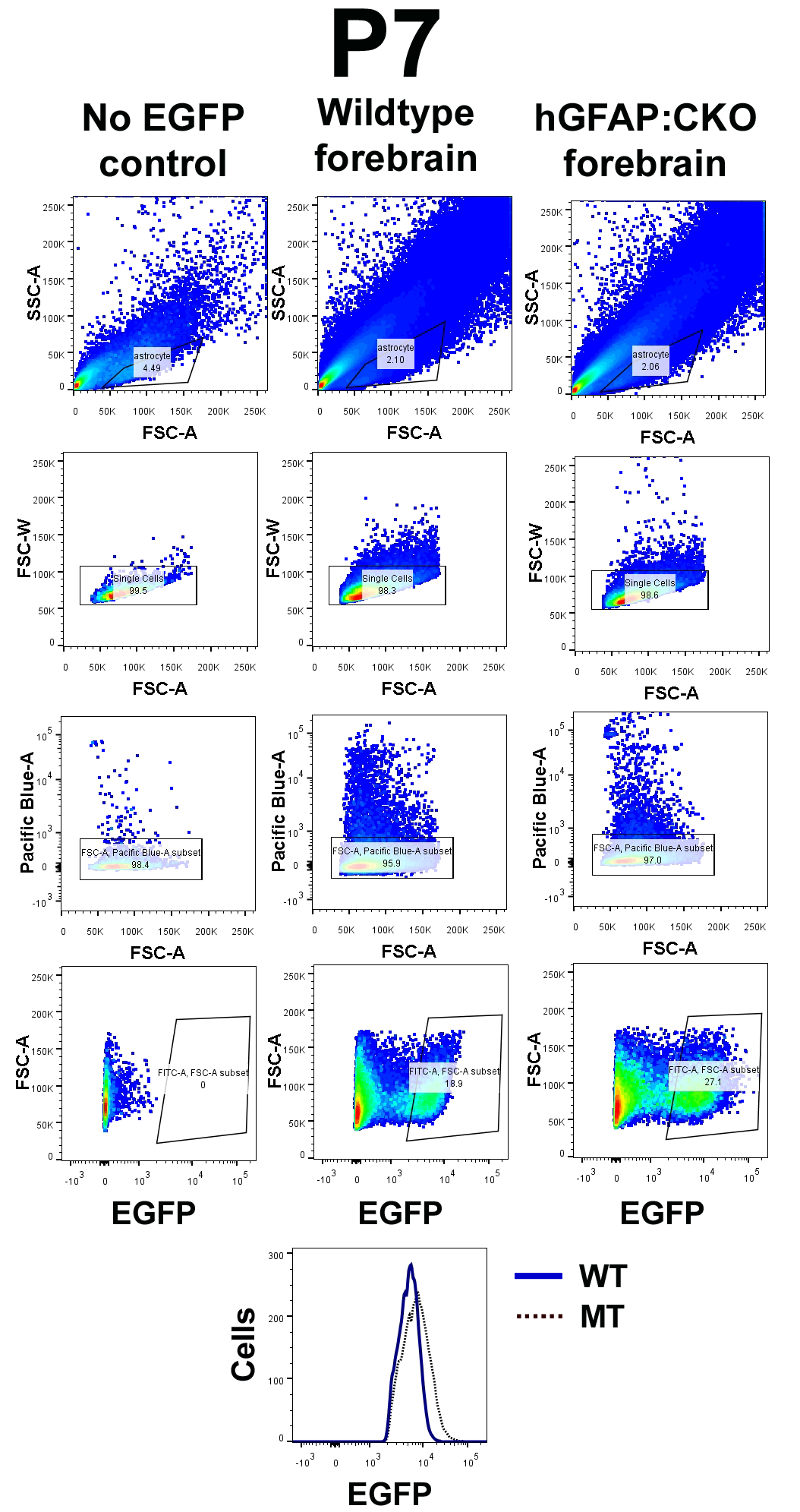

Supplement: S10 Fig — (TIF) [file pone.0126667.s010.tif]

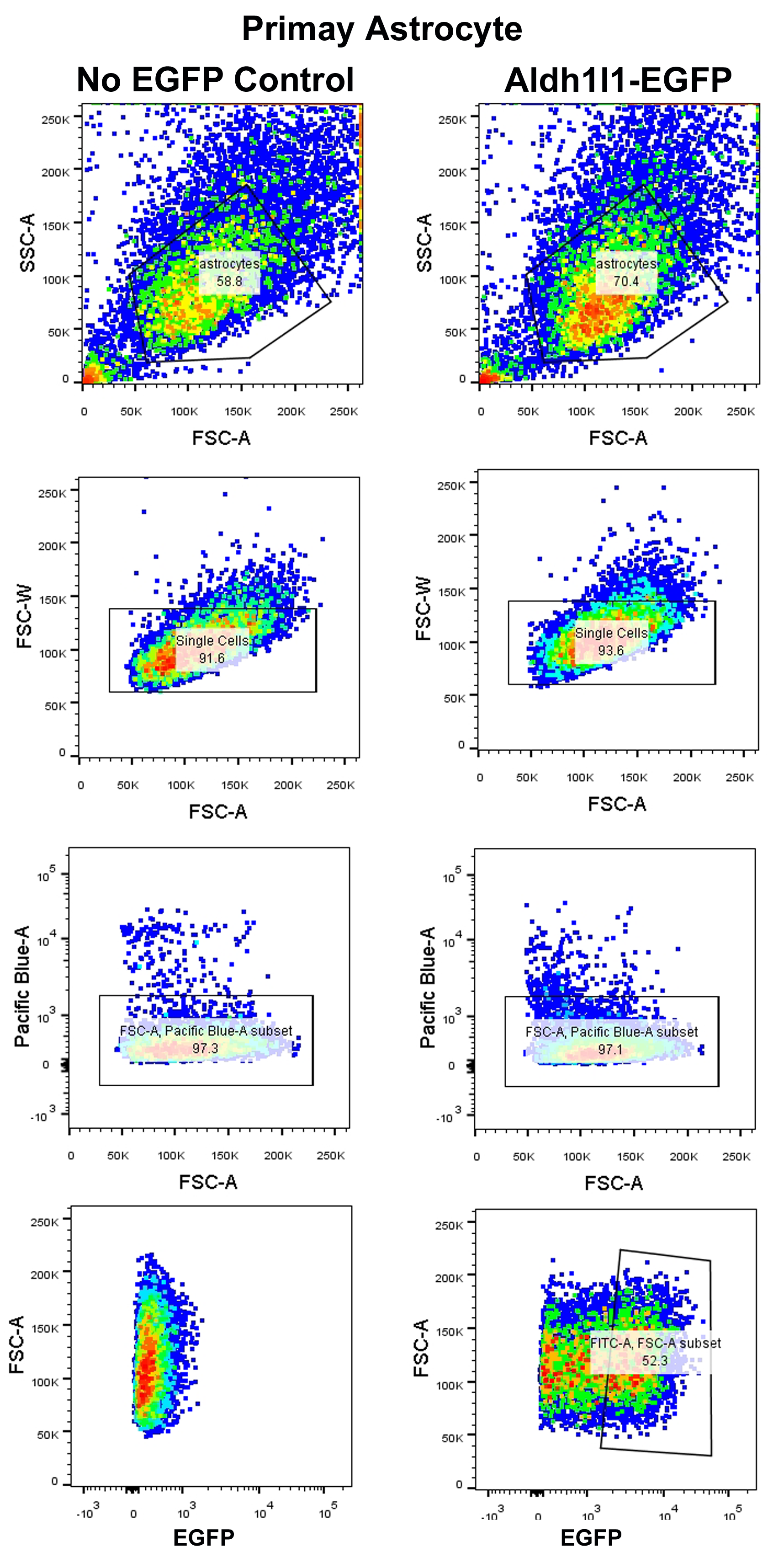

Supplement: S11 Fig — (TIF) [file pone.0126667.s011.tif]
